# Risk factors for poor prognosis in ANCA-associated vasculitis with interstitial lung disease: a systematic review and meta-analysis

**DOI:** 10.1007/s10067-025-07378-z

**Published:** 2025-02-26

**Authors:** Xing He, Weiwei Yuan, Yahui Yang, Jiaqi Ji, Xixi Chen

**Affiliations:** 1 Department of Pulmonary and Critical Care Medicine, West China Hospital, Sichuan University, Chengdu, China; 2 State Key Laboratory of Respiratory Health and Multimorbidity, West China Hospital, Sichuan University, Chengdu, China; 3 School of Medicine, University of Electronic Science and Technology of China, Chengdu, China; 4 Department of Pulmonary and Critical Care Medicine, School of Medicine, Sichuan Provincial People’S Hospital, University of Electronic Science and Technology of China, Chengdu, China; 5 Department of Rheumatology and Immunology, Sichuan Provincial People’S Hospital, University of Electronic Science and Technology of China, Chengdu, China; 6 Department of Rheumatology and Immunology, Qionglai Medical Center Hospital, Chengdu, China

**Keywords:** Antineutrophil cytoplasm antibody-associated vasculitis, Interstitial lung disease, Poor prognosis, Mortality

## Abstract

**Objective:**

Antineutrophil cytoplasm antibody-associated vasculitis (AAV) with interstitial lung disease (AAV-ILD) is the main manifestation of AAV involving the lung, further increasing the risk of poor prognosis in patients with AAV. This study aimed to investigate the risk factors associated with mortality in patients with AAV-ILD.

**Methods:**

In Web of Science, PubMed, Embase and Scopus databases, a comprehensive search was performed for English studies on AAV and ILD published from inception date until May 17, 2024. Hazard ratios (HR) and 95% confidence intervals (CI) of mortality-related risk factors in AAV-ILD were collected, and subgroup analyses were carried out based on different candidate risk factors. Cochran's Q statistic and inconsistency value were utilized to assess the heterogeneity of included studies. Sensitivity analysis was executed using one-by-one elimination method, and publication bias was evaluated with Egger's test and the trim-and-fill method.

**Results:**

A total of 654 patients with AAV-ILD in eight studies were included for the pooled analysis of mortality risk factors. The results showed that age (HR = 1.06, 95%CI: 1.04, 1.08), ever smoker (HR = 1.61, 95%CI: 1.13, 2.29), usual interstitial pneumonia pattern (HR = 2.07, 95%CI: 1.43, 3.00), acute exacerbation (HR = 2.73, 95%CI: 1.70, 4.40) and microscopic polyangiitis (HR = 4.03, 95%CI: 1.70, 9.55) were associated with an increased risk of AAV-ILD mortality. Conversely, percent predicted forced vital capacity (HR = 0.97, 95%CI: 0.96, 0.99) and immunosuppressant for induction (HR = 0.40, 95%CI: 0.28, 0.58) were associated with a reduced risk of AAV-ILD mortality. Male (HR = 1.27, 95%CI: 0.90, 1.80), nervous system involvement (HR = 0.99, 95%CI: 0.65, 1.52), renal involvement (HR = 1.24, 95%CI: 0.97, 1.95) and five factor score ≥ 1 (HR = 1.00, 95%CI: 0.67, 1.48) showed no significant correlation with mortality risk in patients with AAV-ILD. Heterogeneity test indicated no significant heterogeneity among the pooled studies. The results of sensitivity analysis, Egger's test and the trim-and-fill method revealed that the pooled findings were stable and reliable.

**Conclusion:**

The pooled analyses demonstrated that age, ever smoker, usual interstitial pneumonia pattern, acute exacerbation and microscopic polyangiitis were risk factors for mortality in patients with AAV-ILD, while percent predicted forced vital capacity and immunosuppressant therapy for induction serve as protective factors against mortality. A systematic understanding of the risk factors for AAV-ILD may provide clues for developing effective interventions and managements to improve poor prognosis in patients.

**Table:** 

**Key Points** • *Increase of age, ever smoker, usual interstitial pneumonia pattern, acute exacerbation and microscopic polyangiitis were risk factors for poor prognosis in patients with AAV-ILD.* • *High level of percent predicted forced vital capacity and immunosuppressant therapy for induction serve as protective factors against poor prognosis.* • *Male, nervous system involvement, renal involvement and five factor score ≥ 1 showed no significant correlation with poor prognosis in patients with AAV-ILD.*

**Supplementary Information:**

The online version contains supplementary material available at 10.1007/s10067-025-07378-z.

## Introduction

Antineutrophil cytoplasm antibody (ANCA)-associated vasculitis (AAV) is a group of diseases characterized primarily by small-vessel inflammation and fibrinoid necrosis, accompanied by positive serum ANCA antibodies. AAV encompass multiple types, including microscopic polyangiitis (MPA), granulomatosis with polyangiitis (GPA) and eosinophilic granulomatosis with polyangiitis (EGPA). AAV can affect multiple systems throughout the body, such as the skin, kidneys, nervous system, ears, nose, throat and lungs. AAV involve the lungs in the form of pulmonary nodules, cavitary lesions, pleural effusion, alveolar hemorrhage (AH) and interstitial lung disease (ILD) [1]. Previous studies have found that nearly half of patients with AAV develop AAV-related ILD (AAV-ILD) after diagnosis [2], with ILD present in up to 45% of patients with MPA and 23% of patients with GPA [3]. The one-year and five-year overall survival rates for AAV-ILD patients are 88.2% and 81.0%, respectively [4]. Usual interstitial pneumonia (UIP) is a common ILD subtype of AAV-ILD, and more than half of patients with fibrotic AAV-ILD face a high risk of mortality [5]. Therefore, a systematic understanding of the risk factors associated with AAV-ILD mortality would be beneficial for early intervention and disease management.

The poor prognosis of AAV-ILD is influenced by various factors, including infection, malignancy and disease progression; however, there is currently a lack of substantial research evidence. Some retrospective studies have revealed that mortality in patients with AAV-ILD is related to age, smoking history, percent predicted forced vital capacity (FVC%), UIP pattern, organ involvement and acute exacerbation (AE) [4, 6, 7]. Nevertheless, the conclusions in different studies are not completely consistent, and some results remain controversial due to lack of pooled evidence.

Therefore, we aimed to identify the risk factors associated with AAV-ILD mortality by pooled analysis, further enhancing the understanding of relevant factors for poor prognosis in patients with AAV-ILD.

## Materials and methods

This study was conducted in accordance with the Preferred Reporting Project for Systematic Review and Meta-Analysis (PRISMA) guidelines and was registered at INPLASY (http://INPLASY.com)(registration No: INPLASY2024100007).

### Search strategy

A comprehensive search was carried out for English studies published from inception date until May 17, 2024 in Web of Science, PubMed, Embase and Scopus databases. The search terms were as follows: “Interstitial Lung Disease”, “Anti-Neutrophil Cytoplasmic Antibody-Associated Vasculitis”, “microscopic polyangiitis”, “granulomatosis with polyangiitis”, “Churg-Strauss Syndrome”, “Eosinophilic granulomatous polyvasculitis”, “ILD”, “MPA”, “GPA”, “EGPA”, etc. (Supplementary Table 1).

### Eligibility criteria

The criteria for inclusion were as follows: (1) prospective or retrospective studies; (2) the diagnosis of AAV was based on the 2012 Chapel Hill Consensus Conference criteria [8]; (3) the diagnosis of ILD was on the basis of existing clinical guidelines [9, 10], medical history, examination data and multidisciplinary discussion when necessary; (4) mortality risk factors in AAV-ILD were identified by Cox proportional hazards regression model; (5) English literatures.

The criteria for exclusion were as follows: (1) duplicate literatures; (2) case report, conference abstract, review or meta-analysis, animal or cell study, comment or letter, etc.; (3) studies unrelated to AAV-ILD; (4) hazard ratios (HR) and 95% confidence intervals (CI) could not be obtained; (5) inability to extract data; (6) non-English literatures.

### Variable selection and subgroup analysis

Risk factors identified by univariate Cox proportional hazards regression analysis were included in the pooled analysis, provided that at least three included studies could extract relevant data. Subgroup analyses were performed for different risk factors. Unlike meta-analyses defined narrowly, pooled analyses can only be performed when the included studies employed identical study designs and statistical models, and when their respective populations were homogeneous. Due to significant heterogeneity among subgroups, no pooled analysis of risk factors was conducted across subgroups.

### Quality assessment (risk of bias) and data extraction

WY and YY independently screened all studies, with conflicts resolved by an arbitrator (CX). The Newcastle–Ottawa Scale (NOS) was applied for the quality of the included literatures by a semi-quantitative scoring system, with a maximum score of 9 stars [11]; ratings of 1–3 stars, 4–6 stars and 7–9 stars were defined as low, medium, and high quality, respectively. The literature quality assessment was independently completed by XH and JJ. Relevant data were extracted by WY and YY, covering author’s surname, publication year, country, study type, study object, age, male, proportion of smokers, FVC%, UIP pattern, AE, follow-up time, sample size, number of deaths, risk factor, HR and 95%CI.

### Data synthesis

HR and 95%CI of risk factors were collected as statistical effect sizes. The Cochran's Q statistic and inconsistency value (I^2^) was used for heterogeneity analysis. If *P* < 0.05 or I^2^ ≥ 50%, indicating significant heterogeneity, a random effects model and DerSimonian-Laird (DL) method were employed for pooled analysis; otherwise, a fixed effects model and inverse variance (IV) method were used. Subgroup analyses were executed for different risk factors. Excluding one category of study at a time method was utilized for sensitivity analysis. If there was no significant effect on the results after excluding one study, our findings were considered stable and reliable. Publication bias was detected by Egger's test, and the reliability of the studies was judged with a trim-and-fill method. Statistical analyses were computed using the meta package in Stata 16.0 software, with statistical significance at *P* < 0.05.

## Result

**Fig. 1:**
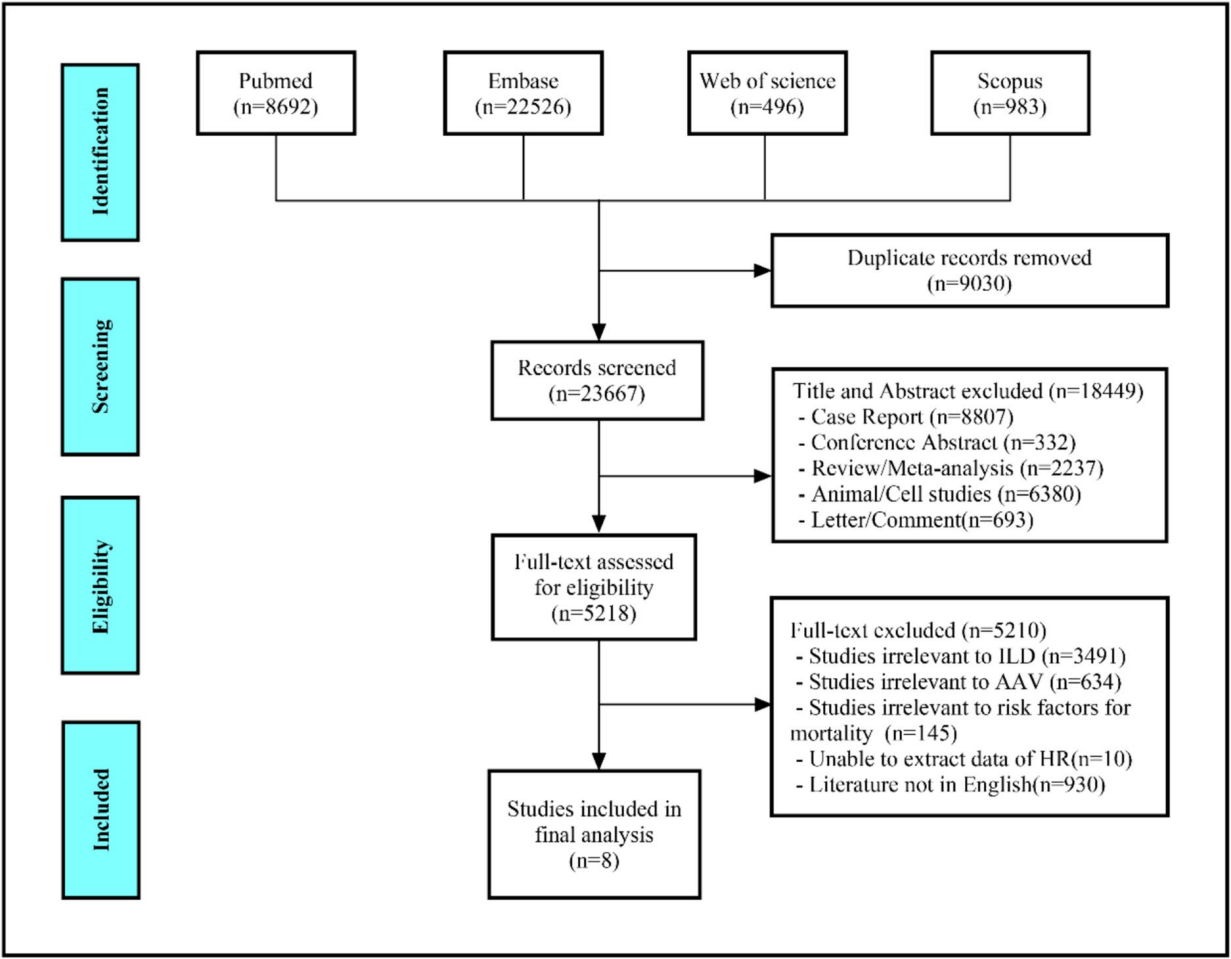
Diagram of the preferred reporting items for systematic review and meta-analysis (PRISMA)

As shown in Fig. 1, after a rigorous evaluation, eight studies involving 654 patients were ultimately eligible for pooled analysis. All included studies were retrospective. The countries involved in the studies were China (*n* = 4), Japan (*n* = 2), South Korea (*n* = 1) and France and Belgium (*n* = 1). The risk factors of AAV-ILD mortality were analyzed, such as age [4, 12–15], male [4, 6, 13, 14], ever smoker [4, 6, 7, 13, 14], FVC% [4, 13, 14, 16], UIP pattern [6, 7, 13–15], AE [4, 6, 7, 13], MPA [4, 13, 15], nervous system involvement (NI) [4, 6, 7, 14], renal involvement (RI) [4, 6, 7, 14], five factor score (FFS) ≥ 1 [4, 6, 14] and immunosuppressant for induction(IFI) [6, 7, 12, 14]. The characteristics of included studies are presented in (Table 1). According to the NOS results, all eight studies were of high quality (Supplementary Table 2).

**Table 1:** Clinical characteristics of AAV-ILD in each included study

No	Author, year	Country	Study type/Object	Age (year)	Male *n* (%)	Ever smoker *n* (%)	FVC%	AAV Subtype *n* (%)	ILD Subtype *n* (%)	AE n(%)	Follow-up time (month)	nTotal/nDeath	Risk factor	HR (95% CI)	Individual level data available
1	Maillet T, et al.2019 [6]	France and Belgium	retrospective (AAV-ILD)	66 (56–74)	34 (55)	36 (59)	NA	MPA: 53 (85) GPA: 9 (15)	UIP: 38 (61) NSIP: 24 (39)	15(25)	40.5 (21–68)	62/19	male	1.771(0.69–4.57)	not given
													ever smoker	2.21(0.86–5.68)	
													UIP pattern	5.49(1.41–8.87)	
													AE	5.01(1.54–16.36)	
													NI	2.42(0.91–6.44)	
													RI	0.92(0.35–2.44)	
													FFS ≥ 1	1.82(0.74–4.49)	
													IFI	1.67(0.36–7.73)	
2	Yang S, et al 2022 [12]	China	retrospective (AAV-ILD)	70.2 ± 9.8	58 (57.4)	57 (56.4)	88.1 (76.9–107.4)	NA	NA	NA	22.7 (2.8–51.2)	101/NA	age	1.042(1.009–1.076)	make request
													IFI	0.324(0.195–0.537)	
3	Matsuda S, et al. 2021 [16]	Japan	retrospective (MPA-ILD)	76 (70–80)	23 (49)	27 (57.5)	NA	MPA: 47 (100)	NA	1(2.1)	NA	47/12	FVC%	0.95(0.9–0.99)	make request
4	Zhou P, et al. 2021 [4]	China	retrospective (AAV-ILD)	68 (62–74)	110 (53.9)	85 (41.7)	81.5 ± 19.8	MPA: 186 (91.2) GPA: 15 (7.4) EGPA: 3 (1.5)	UIP: 152 (74.5) NSIP: 39 (19.1) OP: 3 (1.5) Unclassified: 10 (4.9)	3(1.5)	40 (17–75)	204/44	age	1.545(1.010–2.364)	make request
													male	0.84(0.44–1.604)	
													ever smoker	1.494(0.754–2.959)	
													FVC%	1.015(0.748–1.377)	
													AE	1.586(0.741–3.393)	
													MPA	4.135(0.562–30.445)	
													NI	1.601(0.744–3.446)	
													RI	0.956(0.427–2.139)	
													FFS ≥ 1^*^	1.162(0.573–2.355)	
													FFS ≥ 1^#^	0.595(0.217–1.632)	
5	Zhang Y, et al. 2023 [7]	China	retrospective (MPA-ILD)	72.03 ± 11.19	23 (62.2)	16 (43.24)	71.82 ± 17.8	MPA: 37 (100)	UIP: 20 (54.05) NSIP: 11 (29.73) Unclassified: 6 (16.22)	NA	25 (10.5–45.5)	37/15	male	1.52(0.55–4.17)	make request
													ever smoker	2.27(0.78–6.62)	
													UIP pattern	2.54(0.92–7.04)	
													AE	2.84(0.98–8.21)	
													NI	0.82(0.29–2.33)	
													RI	1.24(0.45–3.42)	
													IFI	0.23(0.083–0.63)	
6	Kim MJ, et al. 2023 [13]	South Korea	retrospective (MPA-ILD)	62.7 ± 9.8	23 (59)	21 (53.8)	77 ± 15.3	MPA: 39 (100)	UIP-like: 31(79.5) Non-UIP-like: 8 (20.5)	7(17.9)	72 (44–117)	39/20	age	1.081(1.018–1.148)	make request
													male	2.294(0.871–6.039)	
													ever smoker	1.767(0.714–4.374)	
													FVC%	0.993(0.968–1.018)	
													UIP pattern	0.98(0.349–2.755)	
													AE	4.203(1.616–10.933)	
													MPA	3.072(0.392–24.075)	
7	Hozumi H, et al. 2021 [14]	Japan	retrospective (MPA-ILD)	73.8 ± 8.7	56 (66.7)	57 (67.9)	84.8 ± 17	MPA:84 (100)	UIP: 33 (39.3) Probable UIP: 14 (16.7) Indeterminate for UIP 11 (13.1) Alternative 26 (30.9)	16 (19.1)	43.9 ± 40.1	84/51	Age	1.04(1.01–1.08)	not given
													male	1.18(0.66–2.23)	
													ever smoker	1.29(0.72–2.44)	
													FVC%	0.96(0.94–0.98)	
													UIP pattern	1.46(0.83–2.55)	
													NI	0.99(0.45–2.59)	
													RI	0.74(0.34–1.46)	
													FFS ≥ 1	0.78(0.4–1.53)	
													IFI	0.5(0.3–1.02)	
8	Sun X, et al. 2021 [15]	China	retrospective (AAV-ILD)	60 (51–66)	36 (45)	NA	81.85 ± 18.42	MPA: 31 (38.8) Non-MPA: 49 (61.2)	UIP: 7 (8.75) NSIP: 51 (63.75) Unclassified: 22 (27.5)	NA	40 (27–58)	80/15	age	1.075(1.011–1.143)	make request
													UIP pattern	3.264(1.203–8.858)	
													MPA	4.31(1.464–12.692)	

According to the study results: ^*^: FFS = 1, ^#^: FFS ≥ 2 *NA* Not required; *FVC%* Percent predicted forced vital capacity; *UIP* Usual interstitial pneumonia; *AE* Acute exacerbation; *HR* Hazard ratio; *CI* Confidence interval; *AAV* Antineutrophil cytoplasm antibody-associated vasculitis; *MPA* Microscopic polyangiitis; *GPA* Granulomatosis with polyangiitis; *EGPA* Eosinophilic granulomatosis with polyangiitis; *ILD* Interstitial lung disease; *UIP* Usual interstitial pneumonia; *NSIP* Nonspecific interstitial pneumonia; *OP* Organizing pneumonia; *NI* Nervous system involvement; *RI* Renal involvement; *FFS* Five factor score; *IFI* Immunosuppressant for induction

### Effects of age, male and ever smoker on AAV-ILD mortality

**Fig. 2:**
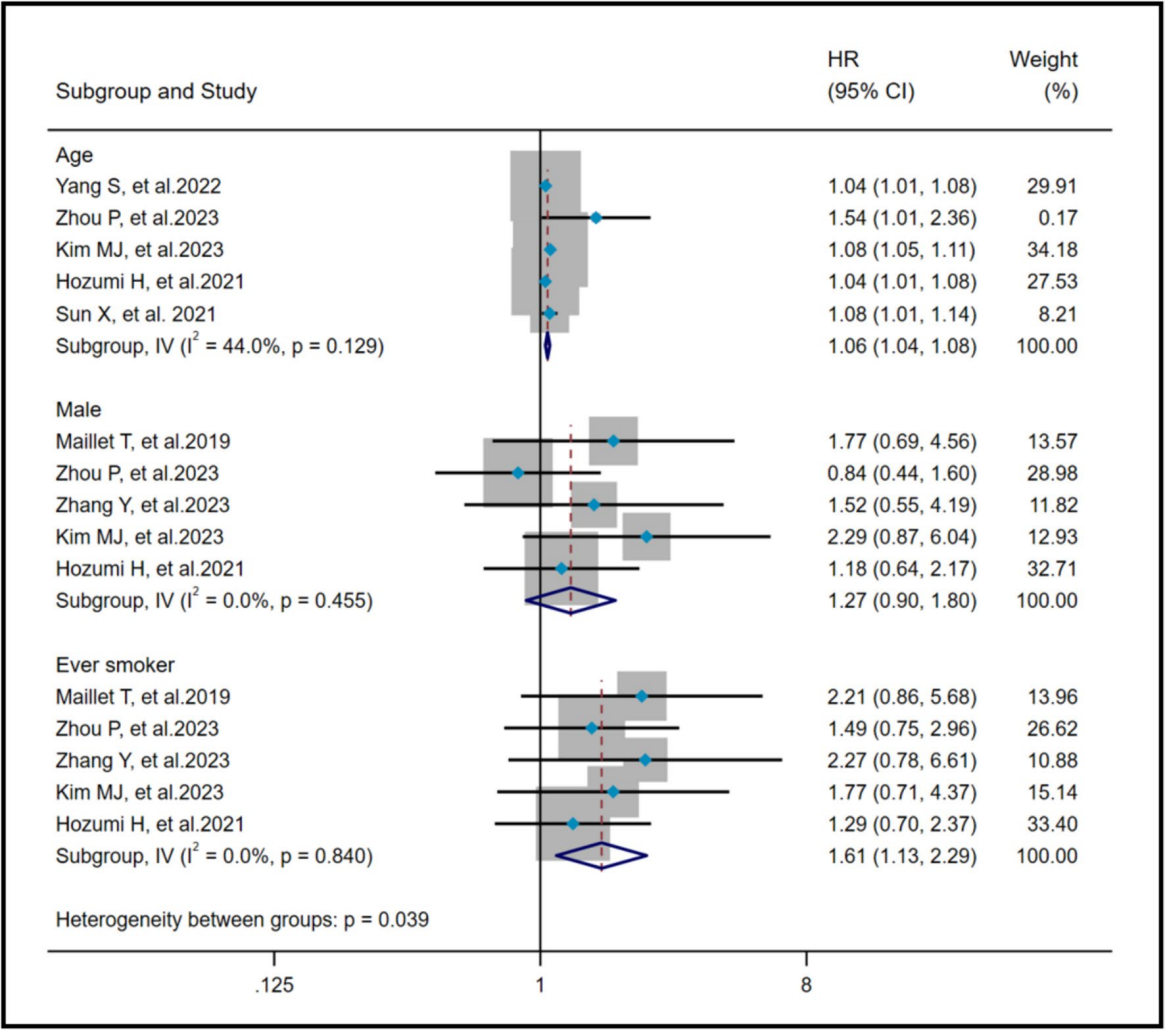
Pooled hazard ratio of age, male and ever smoker on poor prognosis in AAV-ILD

The heterogeneity results indicated that among the risk factors of poor prognosis, age (I^2^ = 44%, *P* = 0.129), male (I^2^ = 0%, *P* = 0.455) and ever smoker (I^2^ = 0%, *P* = 0.84) showed no significant heterogeneity. A fixed effects model and IV method were employed for analysis. Pooled results revealed that age (HR = 1.06, 95%CI: 1.04, 1.08, *P* < 0.001) and ever smoker (HR = 1.61, 95%CI: 1.13, 2.29, *P* = 0.008) were risk factors for AAV-ILD mortality, whereas male (HR = 1.27, 95%CI: 0.90, 1.80, *P* = 0.18) has no effect on AAV-ILD mortality (Fig. 2).

### Effects of FVC%, UIP pattern and AE on AAV-ILD mortality

**Fig. 3:**
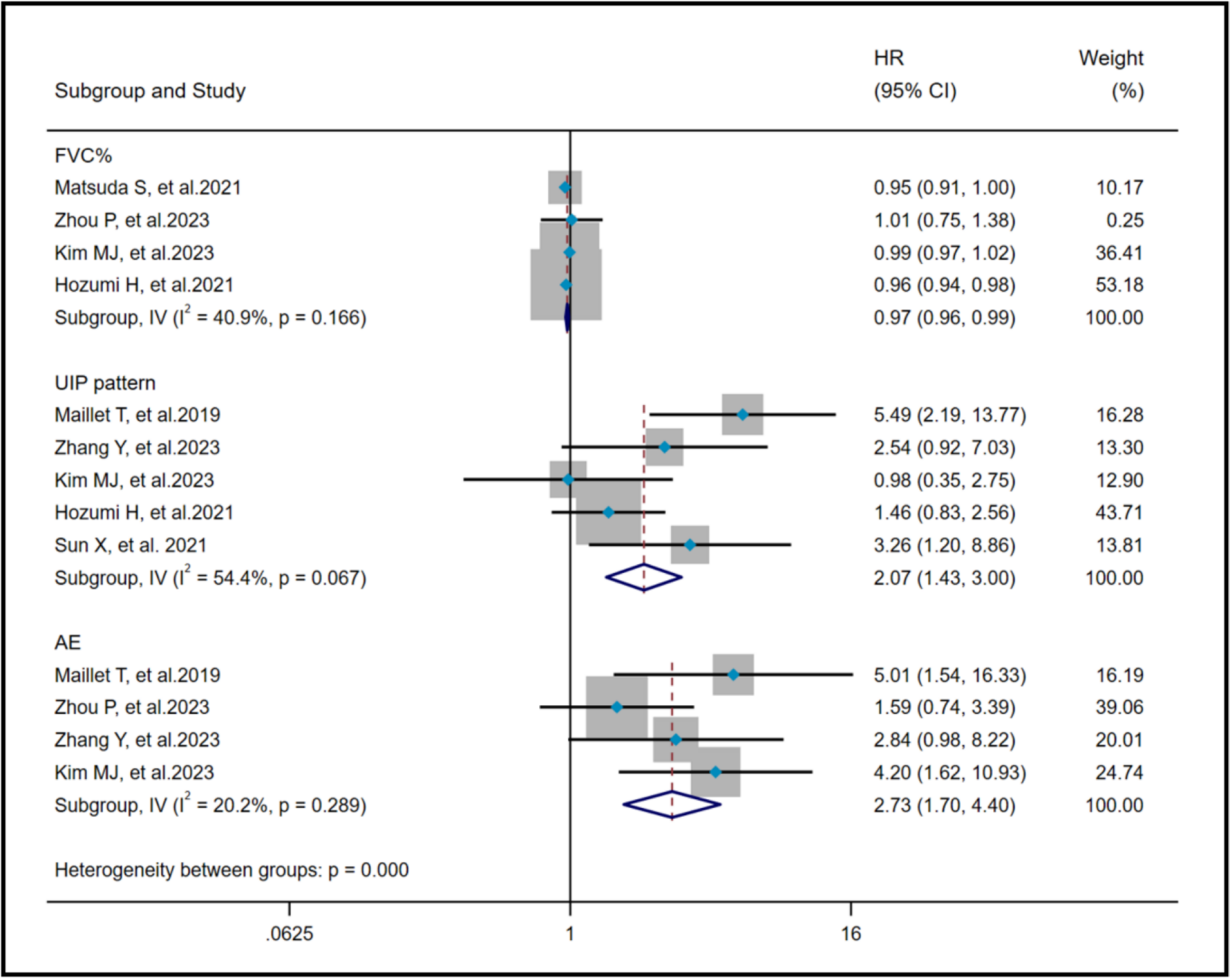
Pooled hazard ratio of FVC%, UIP pattern and AE on poor prognosis in AAV-ILD

The heterogeneity results showed that among the risk factors of poor prognosis, FVC% (I^2^ = 40.9%, *P* = 0.166), UIP pattern (I^2^ = 54.4%, *P* = 0.067) and AE (I^2^ = 20.2%,* P* = 0.289) showed no significant heterogeneity. A fixed effects model and IV method were used, with pooled results indicating that UIP pattern (HR = 2.07, 95%CI: 1.43, 3.00, *P* < 0.001) and AE (HR = 2.73, 95%CI: 1.70, 4.40, *P* < 0.001) were risk factors for AAV-ILD mortality, while FVC% (HR = 0.97, 95%CI: 0.96, 0.99, *P* < 0.001) served as a protective factor against AAV-ILD mortality (Fig. 3).

### Effects of MPA, NI and RI on AAV-ILD mortality

**Fig. 4:**
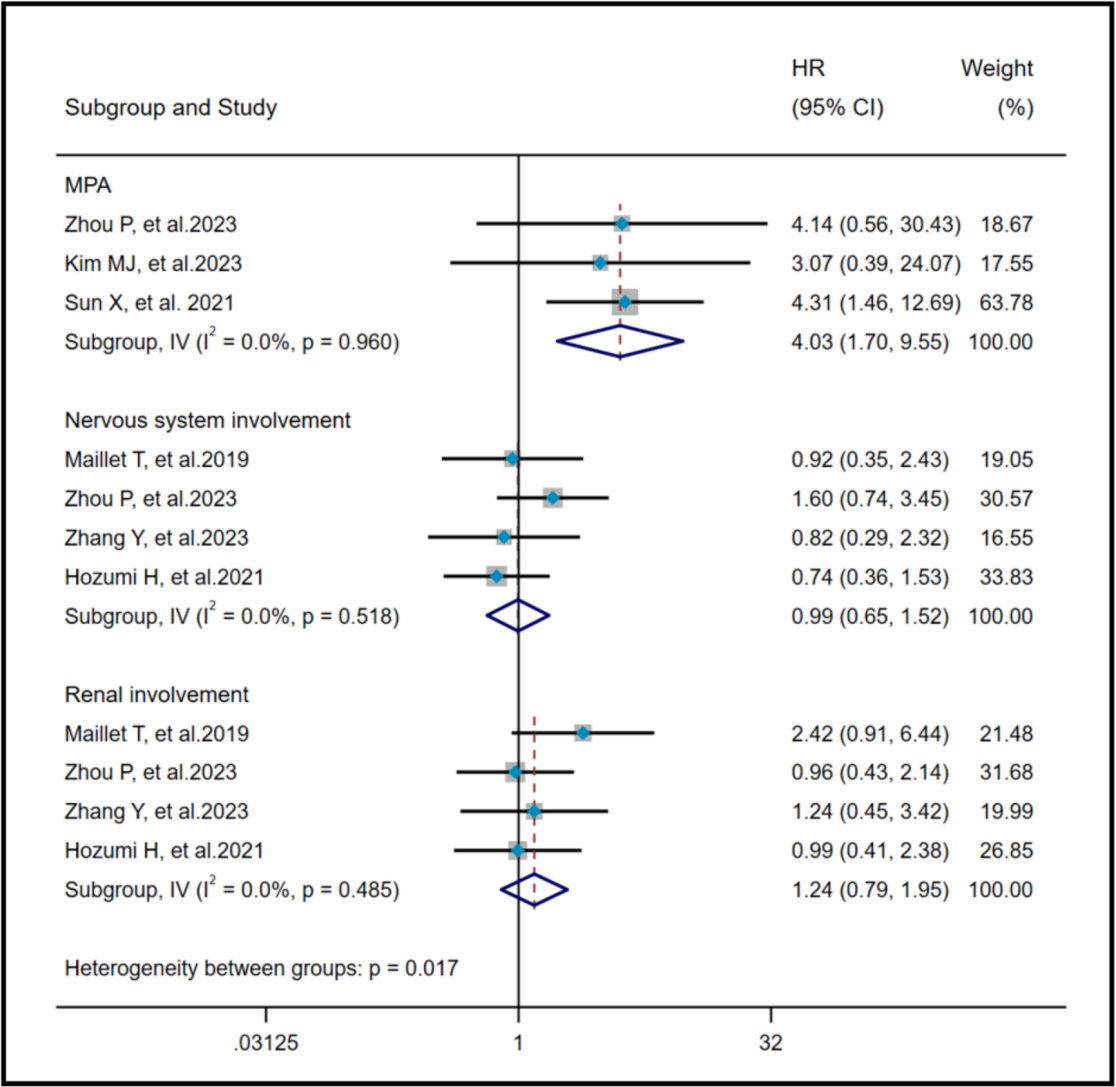
Pooled hazard ratio of MPA, NI and RI on poor prognosis in AAV-ILD

The heterogeneity results presented that there was no significant heterogeneity in MPA (I^2^ = 0%, *P* = 0.96), NI (I^2^ = 0%, *P* = 0.518) and RI (I^2^ = 0%, *P* = 0.485). Using a fixed effects model and IV method, pooled results demonstrated that MPA (HR = 4.03, 95%CI: 1.70, 9.55, *P* = 0.002) was a risk factor for AAV-ILD mortality, whereas NI (HR = 0.99, 95%CI: 0.65, 1.52, *P* = 0.975) and RI (HR = 1.24, 95%CI: 0.97, 1.95, *P* = 0.351) had no effect on AAV-ILD mortality (Fig. 4).

### Effects of FFS ≥ 1 and immunosuppressant for induction on AAV-ILD mortality

**Fig. 5:**
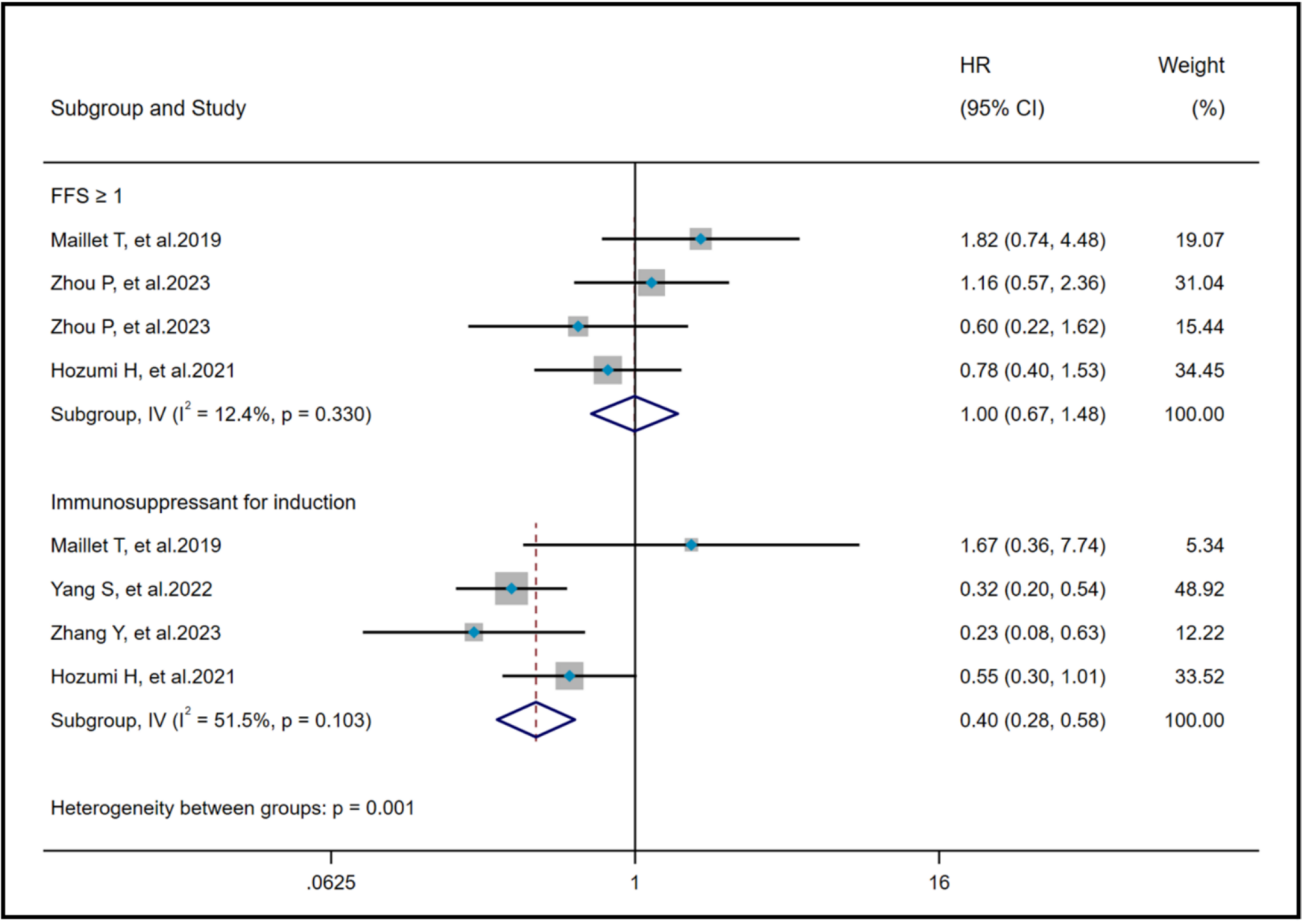
Pooled hazard ratio of FFS ≥ 1 and IFI on poor prognosis in AAV-ILD

Among the risk factors of poor prognosis, the results of FFS ≥ 1 (I^2^ = 12.4%, *P* = 0.33) and IFI (I^2^ = 51.5%, *P* = 0.103) indicated no significant heterogeneity. A fixed effects model and IV method were employed for analysis. Pooled results revealed that IFI (HR = 0.40, 95%CI: 0.28, 0.58, *P* < 0.001) was a protective factor against AAV-ILD mortality, while FFS ≥ 1 (HR = 1.00, 95%CI: 0.67, 1.48, *P* = 0.98) did not affect AAV-ILD mortality (Fig. 5).

### Sensitivity analysis and publication bias

**Fig. 6:**
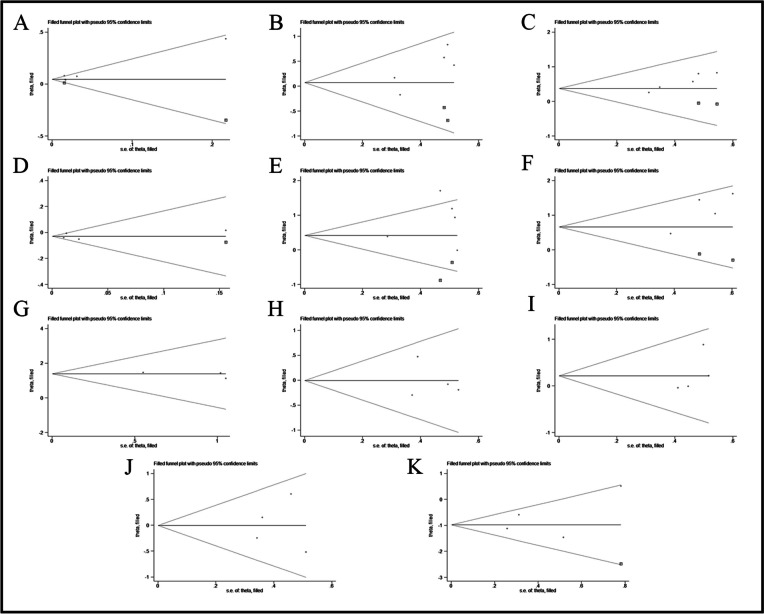
Funnel plots of trim-and-fill analysis for hazard ratio predicting poor prognosis of AAV-ILD (**A**: age, **B**: male, **C**: ever smoker, **D**: FVC%, **E**: UIP pattern, **F**: AE, **G**: MPA, **H**: NI, **I**: RI, **J**: FFS ≥ 1, **K**: IFI)

The sensitivity analysis indicated that our findings were stable (Supplementary Figs. 1–11). There was no significant heterogeneity among the included studies for all factors. Egger's test revealed a potential publication bias related to the risk factor of ever smoker in the included studies (*P* < 0.05) (Table 2). Subsequently, all pooled studies on risk factors associated with AAV-ILD mortality were adjusted by the trim-and-fill method. The analyses for age (*n* = 2), male (*n* = 2), ever smoker (*n* = 2), FVC% (*n* = 1), UIP pattern (*n* = 2), AE (*n* = 2), IFI (*n* = 1), MPA (*n* = 0), NI (*n* = 0), RI (*n* = 0), FFS ≥ 1 (*n* = 0) showed that after supplementing corresponding literatures, the funnel plots were symmetrical and the statistical results remained unchanged. These suggested that there was no publication bias in the included studies (Fig. 6A-K).

**Table 2:** Sensitivity analysis, Egger's test, Metatrim filled study, funnel plot and publication bias of included studies with different subgroups

Risk factors	Sensitivity analysis (excluded study)	Included items	Egger’s test		Metatrim filled study	Funnel Plot	Publication bias
			t	*P*			
Age	0	5	1.38	0.261	2	symmetry	no
Male	0	5	2.39	0.097	2	symmetry	no
Ever smoker	0	5	6.76	0.007	2	symmetry	no
FVC%	0	4	0.04	0.974	1	symmetry	no
UIP pattern	0	5	0.82	0.473	2	symmetry	no
AE	0	4	2.44	0.135	2	symmetry	no
MPA	0	3	−1.08	0.474	0	symmetry	no
NI	0	4	−0.28	0.806	0	symmetry	no
RI	0	4	1.32	0.317	0	symmetry	no
FFS ≥ 1	0	4	0.06	0.958	0	symmetry	no
IFI	0	4	0.72	0.545	1	symmetry	no

*FVC%* Percent predicted forced vital capacity; *UIP* Usual interstitial pneumonia; *AE* Acute exacerbation; *NI* Nervous system involvement; *RI* Renal involvement; *FFS* Five factor score; *IFI* Immunosuppressant for induction

## Discussion

Exploring the risk factors of poor prognosis in patients with AAV-ILD has become a hot topic in recent years. However, some controversial results were observed across different studies. Identifying factors associated with AAV-ILD mortality can provide a more systematic and comprehensive understanding of the disease, and is conducive to the intervention and management strategies. Therefore, we conducted a pooled analysis of potential risk factors for the first time, and demonstrating that age, ever smoker, FVC%, UIP pattern, AE, MPA and IFI were associated with AAV-ILD mortality.

The pooled results indicated that age was a risk factor for AAV-ILD mortality, and the risk of death increased with age. This is likely influenced by multiple factors, such as infection in the course of the disease and disease progression [17]. Patients with smoking history also face a higher risk of death, despite the widespread recognition that smoking leads to deterioration of pulmonary function. Patel A et al. found that ever smokers significantly increased the disease activity of AAV compared to non-smokers, which was associated with renal replacement therapy and the use of immunosuppressants, ultimately leading to poor survival prognosis in patients [18]. It is suggested that the mechanisms by which smoking impacts adverse prognosis of AAV-ILD are complex. Furthermore, our findings showed that AAV-ILD manifested in UIP pattern was also had a high mortality risk, with UIP pattern being related to pulmonary function deterioration and disease progression in ILD [19]. This may be one of the contributing factors for the increased mortality risk. Nurmi HM et al. noted that patients with rheumatoid arthritis-related interstitial lung disease with UIP pattern had significantly higher hospitalization frequency and oxygen therapy rates compared to those with nonUIP pattern [20]. AAV-ILD patients with UIP pattern have a higher mortality risk than those with nonUIP pattern [21], highlighting the need for greater attention to patients with UIP pattern. It is worth mentioning that AH is also a prevalent manifestation of AAV involvement in the lungs and is regarded as one of the potentially fatal outcomes associated with AAV [22]. However, due to strict criteria and the limited number of studies capable of extracting relevant data, we were unable to perform a pooled analysis on AH. It is anticipated that future research will elucidate the relationship between AH and ILD with the poor prognosis in patients with AAV. A study pointed out that 29% of patients with ILD experienced AE during the disease course, and the risk of death was significantly higher than that of patients with nonAE [23]. AE-ILD may be triggered by factors such as infections, mechanical stress and microaspiration [24]. The prognosis of AAV-ILD patients with AE is also poor, but it remains unclear whether the occurrence of AE is related to factors inherent to AAV. MPA is one of the main subtypes of AAV. Kim MJ et al. reported that approximately 50% of patients with MPA-ILD died within six years of follow-up [13]. Respiratory infection, diffuse alveolar hemorrhage and AE make a difference to mortality in patients with MPA-ILD [25]. However, further evidence is lacking regarding the impact of disease activity, major organ involvement and treatment strategies on poor prognosis of MPA.

In our study, a decline in FVC% signified an increased risk of AAV-ILD mortality. The decreased level of FVC%, an important indicator of pulmonary function, was confirmed to be significantly correlated with the mortality in ILD [26], which was consistent with research focusing on idiopathic pulmonary fibrosis [27]. It indicates that FVC% is significantly important for evaluating the prognosis of AAV-ILD. Moreover, immunosuppressants for induction is effective in improving poor prognosis in AAV-ILD patients. The combination of glucocorticoids and cyclophosphamide can extend the survival time of patients with AAV-related pulmonary involvement [7], yet there is a lack of studies on the efficacy of other immunosuppressants, such as rituximab and azathioprine, in the induction treatment of AAV-ILD. Some factors that may not be associated with poor prognosis of AAV-ILD were identified in our pooled analysis. A study by Kwon HC et al. on drug-naïve in patients with AAV confirmed that the mortality rate of male patients was approximately twice that of female patients, accompanied by significantly reduced follow-up times, so male sex was considered as an independent predictor of all-cause mortality in patients with AAV [28]. However, in our study, male sex did not serve as a predictive risk factor for mortality in AAV-ILD patients, possibly because all patients received drug-related treatment, and there was no evidence of increased mortality risk in male patients with other types of ILD. Additionally, RI and NI could not work as relevant factors affecting prognosis in patients with AAV-ILD. Hirayama K et al. demonstrated that ILD significantly increased mortality risk in patients with AAV-related RI [22]. Although patients with AAV-ILD are prone to concurrent RI during the disease course, it is noted that treatment with hormones and immunosuppressants for ILD can simultaneously improve RI and increase survival rate [29, 30]. Therefore, it is believed that concurrent RI may not further elevate the mortality risk in patients with AAV-ILD. Furthermore, Suppiah R et al. verified that AAV-related peripheral NI does not affect pulmonary function and overall mortality rate [31]. It was consistent with our findings, indicating that NI cannot be considered as a risk factor for poor prognosis in AAV-ILD. Finally, an increase in FFS had no impact on the prognosis of AAV-ILD. FFS is used as an assessment tool for adverse prognostic factors in patients with AAV, but it needs to be acknowledged that mortality in AAV-ILD may be more attributable to ILD, including infections from the lung, AE-ILD, fibrosis progression, pulmonary function deterioration and respiratory failure. Since FFS does not comprise these factors, it may not be applicable to the specific population of AAV-ILD.

In fact, exploring the factors associated with poor prognosis in patients with AAV-ILD is a long-term effort, and there are still some important aspects worth developing. On one hand, the relationship between biomarkers with potential diagnostic value and poor prognosis in AAV-ILD is not yet clear, such as Krebs Von den Lungen-6 and surfactant protein D. On the other hand, whether antifibrotic drugs can improve the poor prognosis of AAV-ILD needs more attention in the future.

Our study has some limitations. First, all the included studies were retrospective studies with small sample sizes; The variables incorporated in the regression models of these studies were not entirely consistent, which rendered the results of multi-factor Cox regression non-comparable and difficult to interpret. Consequently, we were unable to perform a pooled analysis of the outcomes from multi-factor Cox regression; additionally, it was essential to interpret pooled results with a risk ratio close to 1 with caution. The candidate risk factors identified from pooled results need to be further clarified by a large sample, prospective and multi-center study. Second, we did not perform pooled analyses of certain potential risk factors presented in some studies, such as Birmingham Vasculitis Activity Score, involvement of the ear, nose, and throat, as well as blood creatinine levels, due to insufficient study evidence available for inclusion in a meta-analysis. Finally, owing to the limited number of studies, we were unable to separately analyze the risk factors for patients with ANCA-ILD regarding MPO + and PR3 + . Our primary objective was to explore the risk factors associated with mortality in AAV-ILD, which necessitated the exclusion of numerous studies focused on other adverse outcomes in this population [32, 33]. Consequently, we did not perform a pooled analysis of prognostic topics such as infection, disease progression, recurrence, and hospitalization among patients with AAV-ILD.

## Conclusion

In summary, our pooled analyses showed that age, ever smoker, FVC%, UIP pattern, AE, MPA and IFI were associated with AAV-ILD mortality. Early intervention and management of risk factors related to AAV-ILD will provide benefits to improve the poor prognosis of patients.

## Supplementary Information

Below is the link to the electronic supplementary material.

Supplementary file1 (DOCX 69.3 MB)

## Data Availability

The original data presented in the study are included in the article/Supplementary Material, further inquiries can be directed to the corresponding author.
