# Supplementary material for: Risk factors for poor prognosis in ANCA-associated vasculitis with interstitial lung disease: a systematic review and meta-analysis
Source: Clin Rheumatol. 2025 Feb 26;44(4):1675–89. doi: 10.1007/s10067-025-07378-z (PMC11993495; doi:10.1007/s10067-025-07378-z)

Supplementary Table 1 Database and Search strategy

| **Database** | **Search strategy** |
| --- | --- |
| **Pubmed**  **(n=8692)** | #1: (((((((((((((Lung Diseases, Interstitial[MeSH Terms]) OR (Diffuse Parenchymal Lung Disease[Title/Abstract])) OR (Interstitial Lung Disease[Title/Abstract])) OR (Lung Disease, Interstitial[Title/Abstract])) OR (Pneumonia, Interstitial[Title/Abstract])) OR (Interstitial Pneumonia[Title/Abstract])) OR (Pneumonias, Interstitial[Title/Abstract])) OR (Pneumonitis, Interstitial[Title/Abstract])) OR (Interstitial Pneumonitis[Title/Abstract])) OR (Pneumonitides, Interstitial[Title/Abstract]))) )) OR (ILD[Title/Abstract])  =101467 items |
|  | #2: (Anti-Neutrophil Cytoplasmic Antibody-Associated Vasculitis[MeSH Terms]) OR (ANCA[Title/Abstract])  =18049 items |
|  | #3: ((Granulomatosis with Polyangiitis[MeSH Terms]) OR (Wegener[Title/Abstract])) OR (GPA[Title/Abstract])  =25977 items |
|  | #4: (((Churg-Strauss Syndrome[MeSH Terms]) OR (Eosinophilic granulomatous polyvasculitis[Title/Abstract])) OR (Allergic Granulomatous Angiitis[Title/Abstract])) OR (Eosinophilic Granulomatous Vasculitis) OR (EGPA[Title/Abstract])  =3875 items |
|  | #5: (Microscopic Polyangiitis[MeSH Terms]) OR (MPA[Title/Abstract])  =49169 items |
|  | #6: #2 OR #3 OR #4 OR #5=81310 items |
|  | #7: #1 AND #6=8692 items |
| **Embase**  **(n=22526)** | #1: 'interstitial lung disease'/exp OR 'lung diseases, interstitial':ab,ti OR 'diffuse parenchymal lung disease':ab,ti OR 'interstitial lung disease':ab,ti OR 'interstitial pneumonia':ab,ti OR 'ild':ab,ti  =156885 items |
|  | #2: 'wegener granulomatosis'/exp OR 'wegener granulomatosis':ab,ti OR 'gpa':ab,ti OR 'granulomatosis with polyangiitis':ab,ti OR 'wegener':ab,ti  =32964 items |
|  | #3:'anca associated vasculitis'/exp OR 'anca associated vasculitis':ab,ti OR 'anca':ab,ti OR 'anti-neutrophil cytoplasmic antibody':ab,ti  =38588 items |
|  | #4: 'churg strauss'/exp OR 'churg strauss':ab,ti OR 'eosinophilic granulomatosis polyangitis':ab,ti OR 'allergic granulomatous angitis':ab,ti OR 'egpa':ab,ti  =7575 items |
|  | #5: 'microscopic polyangiitis'/exp OR 'microscopic polyangiitis':ab,ti OR 'mpa':ab,ti  =53329 items |
|  | #6: #2 OR #3 OR #4 OR #5  =99125 items |
|  | #7: #1 AND #6  =22538 items |
| **Web of science**  **(n=496)** | #1: TS= (Lung Diseases, Interstitial OR Diffuse Parenchymal Lung Disease OR Interstitial Lung Disease OR Interstitial Pneumonia OR ILD)  =41568 items |
|  | #2: TS= (Anti-Neutrophil Cytoplasmic Antibody-Associated Vasculitis OR ANCA OR Eosinophilic granulomatous polyvasculitis OR Churg Strauss Syndrome OR Allergic Granulomatous Angiitis OR Eosinophilic Granulomatous Vasculitis OR EGPA OR Granulomatosis with Polyangiitis OR Wegener OR GPA OR Microscopic Polyangiitis OR MPA)  =350775 items |
|  | #3: #1 AND #2 =496 items |
| **Scopus**  **(n=983)** | #1: TITLE-ABS-KEY ("lung diseases, interstitial" OR "diffuse parenchymal lung disease" OR "interstitial lung disease" OR "interstitial pneumonia" OR "ild")  =51958 items |
|  | #2: TITLE-ABS-KEY ( "Anti-Neutrophil Cytoplasmic Antibody-Associated Vasculitis" OR "ANCA" OR "Eosinophilic granulomatous polyvasculitis" OR "Churg Strauss Syndrome" OR "Allergic Granulomatous Angiitis" OR "Eosinophilic Granulomatous Vasculitis" OR "EGPA" OR "Granulomatosis with Polyangiitis" OR "Wegener" OR "GPA" OR "Microscopic Polyangiitis" OR "MPA" )  =442461 items |
|  | #3:#1 AND #2 =983 items |

Supplementary Table 2 Results of Newcastle-Ottawa quality assessment Scale for each included study

| **Study** | **Selection** | **Comparability** | **Outcome** | **Total** |
| --- | --- | --- | --- | --- |
| Maillet T, et al.2019 | **★★★★** | **★** | **★★★** | **8★** |
| Yang S, et al.2022 | **★★★★** | **★** | **★★** | **7★** |
| Matsuda S, et al. 2021 | **★★★★** | **★★** | **★★★** | **9★** |
| Zhou P, et al. 2021 | **★★★★** | **★** | **★★** | **7★** |
| Zhang Y, et al. 2023 | **★★★★** | **★★** | **★★** | **8★** |
| Kim MJ, et al. 2023 | **★★★★** | **★** | **★★** | **7★** |
| Hozumi H, et al. 2021 | **★★★★** | **★** | **★★★** | **8★** |
| Sun X, et al. 2021 | **★★★★** | **★** | **★★** | **7★** |

Supplementary Figure 1 Plot for the assessment of heterogeneity among the included studies of age on poor prognosis in AAV-ILD through One-by-one elimination method


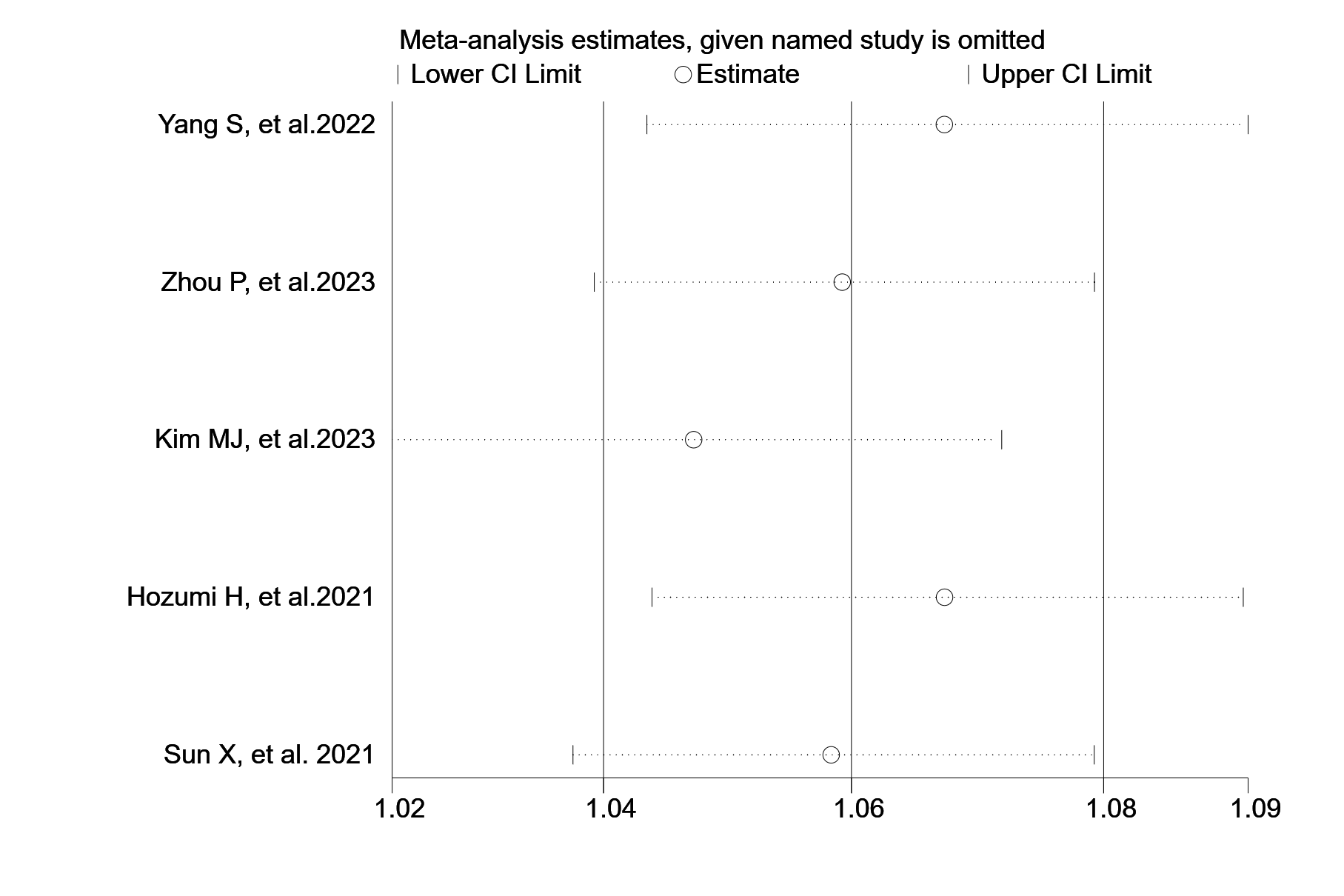


Supplementary Figure 2 Plot for the assessment of heterogeneity among the included studies of male on poor prognosis in AAV-ILD through One-by-one elimination method


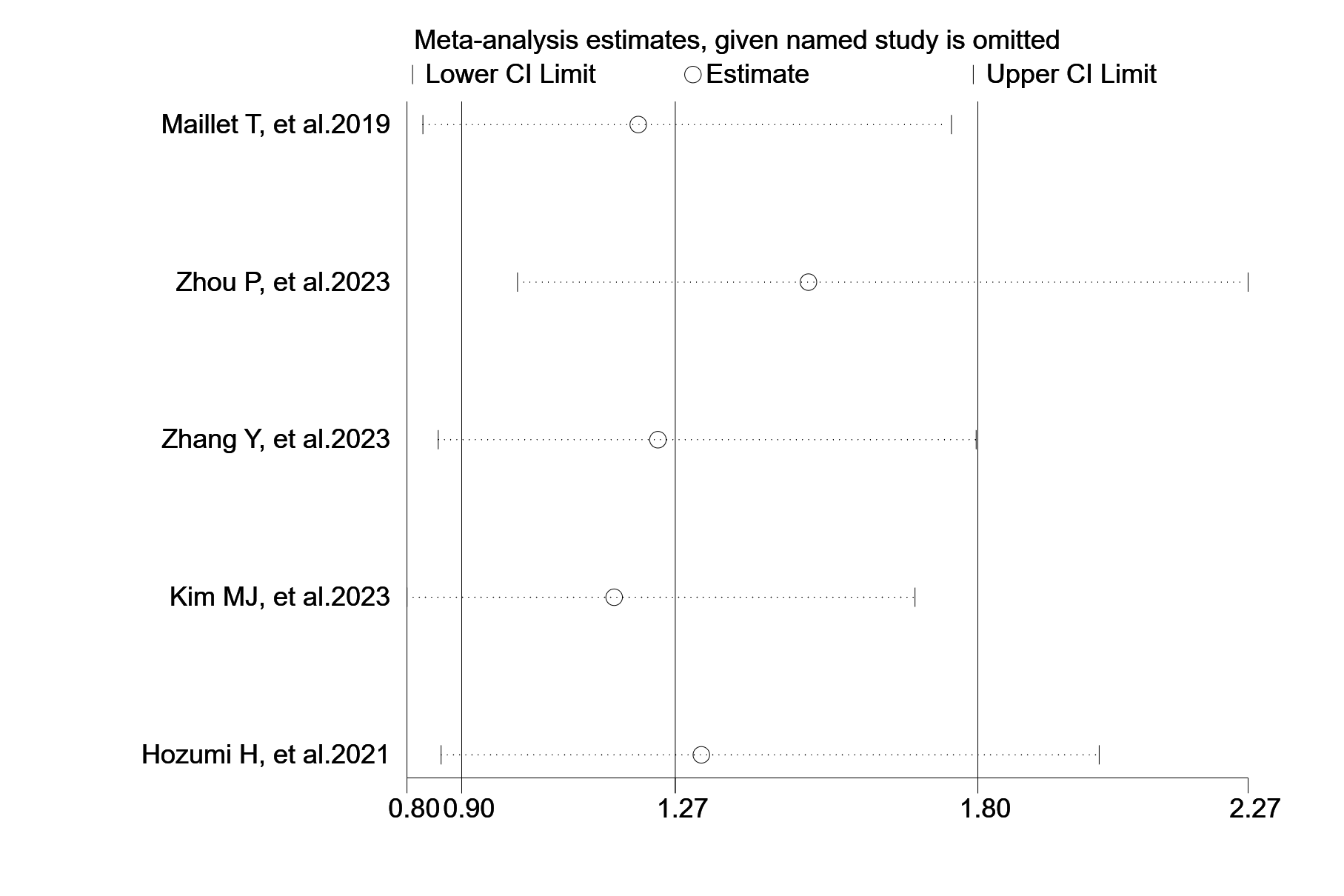


Supplementary Figure 3 Plot for the assessment of heterogeneity among the included studies of ever smoker on poor prognosis in AAV-ILD through One-by-one elimination method


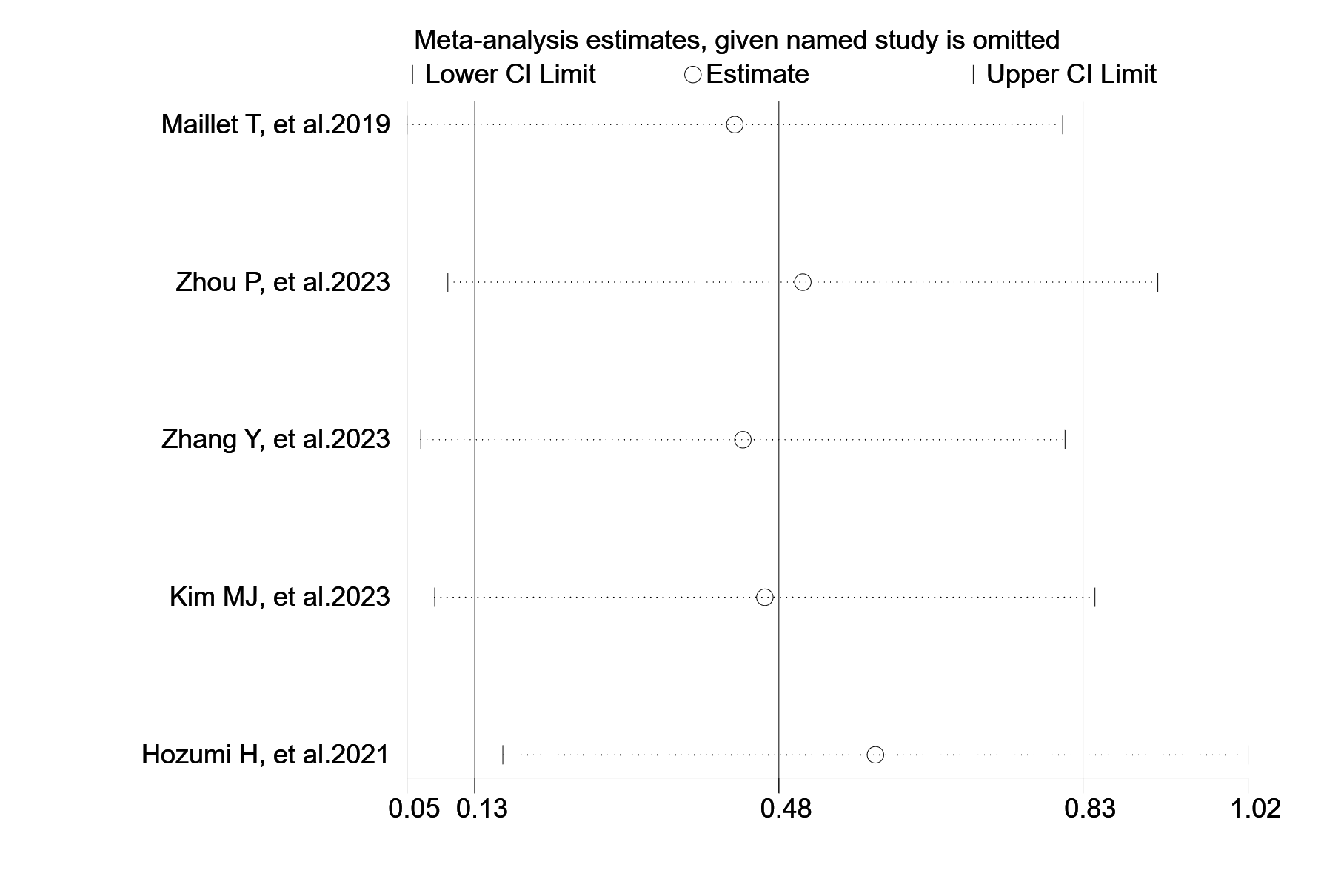


Supplementary Figure 4 Plot for the assessment of heterogeneity among the included studies of FVC% on poor prognosis in AAV-ILD through One-by-one elimination method


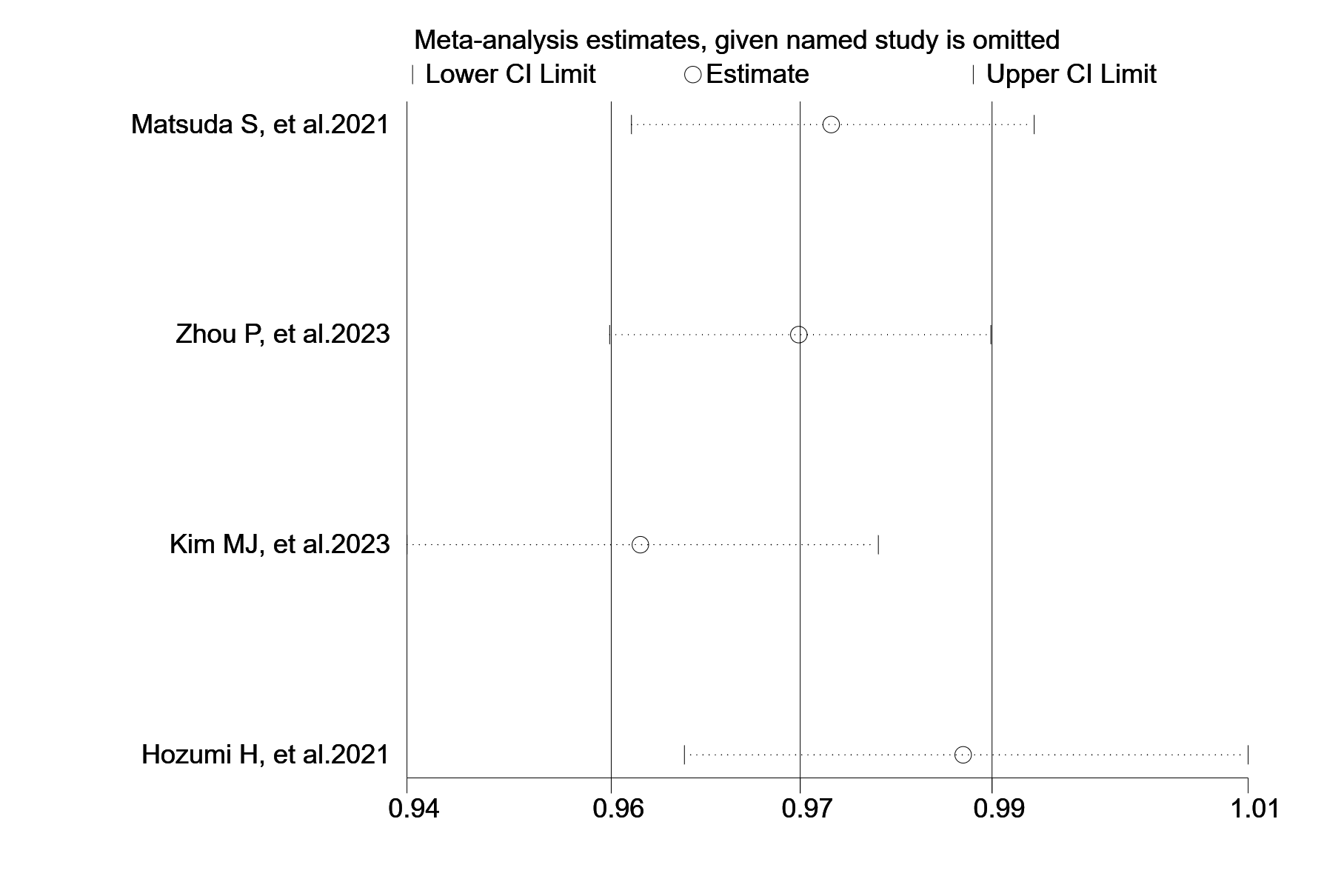


Supplementary Figure 5 Plot for the assessment of heterogeneity among the included studies of UIP pattern on poor prognosis in AAV-ILD through One-by-one elimination method


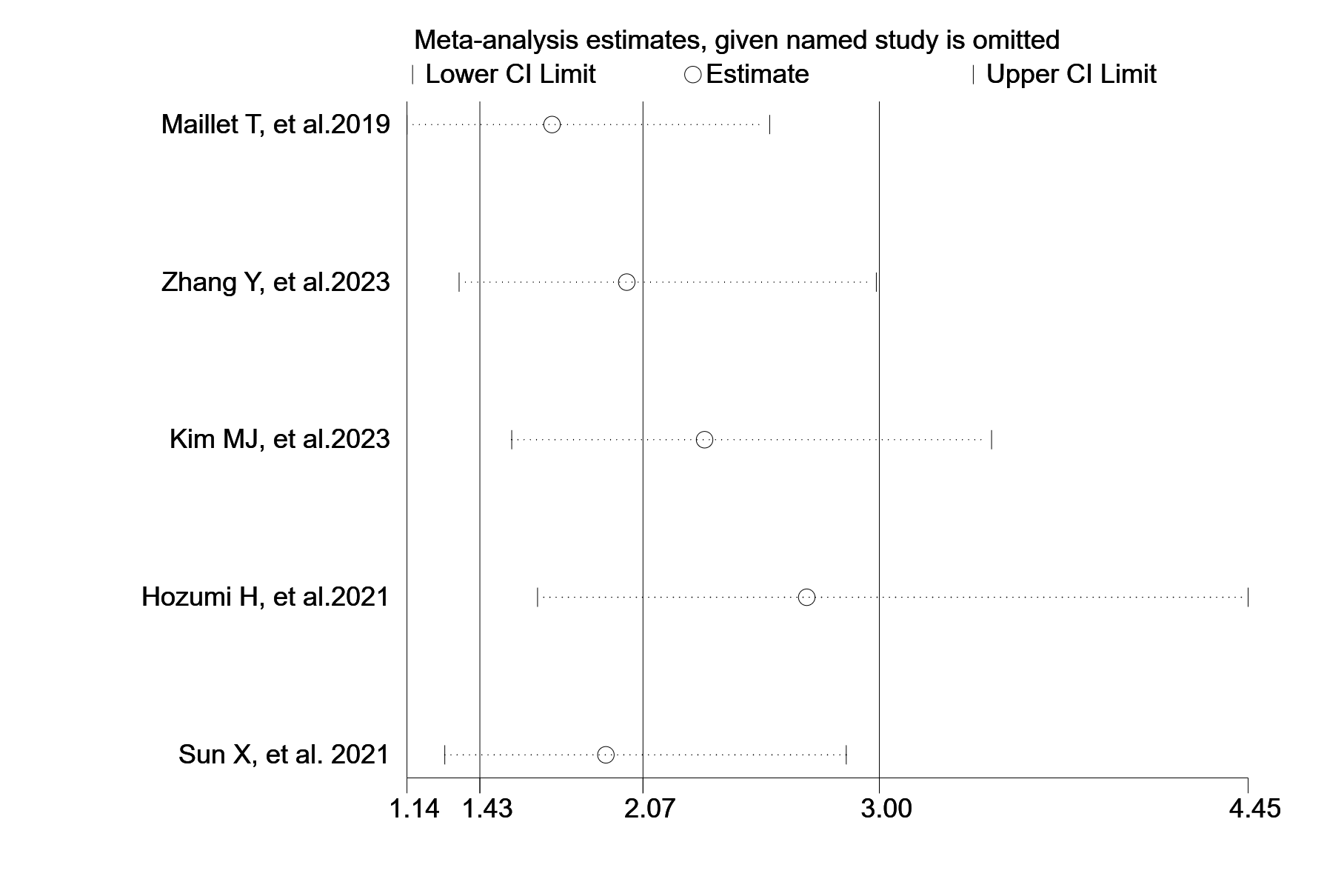


Supplementary Figure 6 Plot for the assessment of heterogeneity among the included studies of AE on poor prognosis in AAV-ILD through One-by-one elimination method


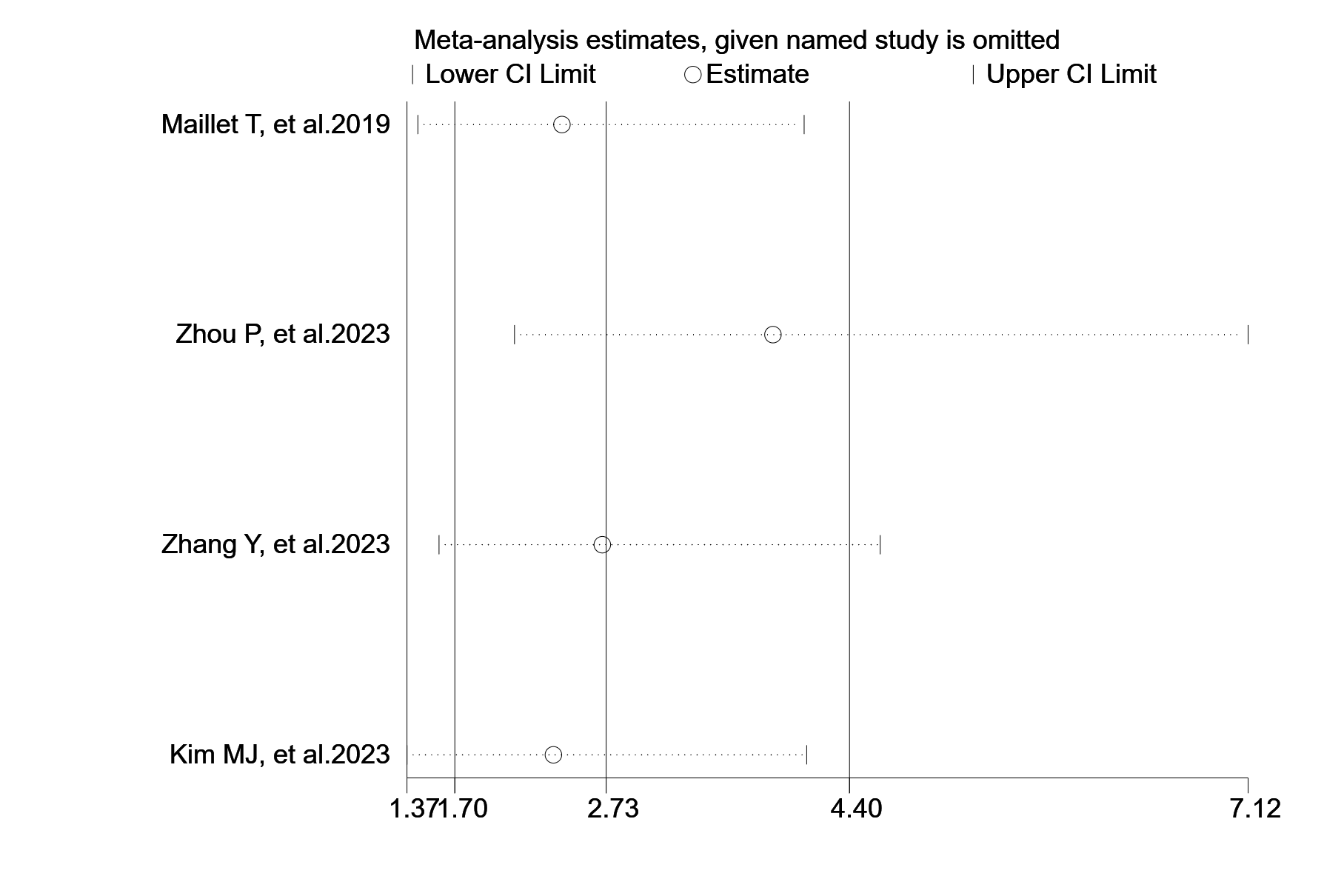


Supplementary Figure 7 Plot for the assessment of heterogeneity among the included studies of MPA on poor prognosis in AAV-ILD through One-by-one elimination method


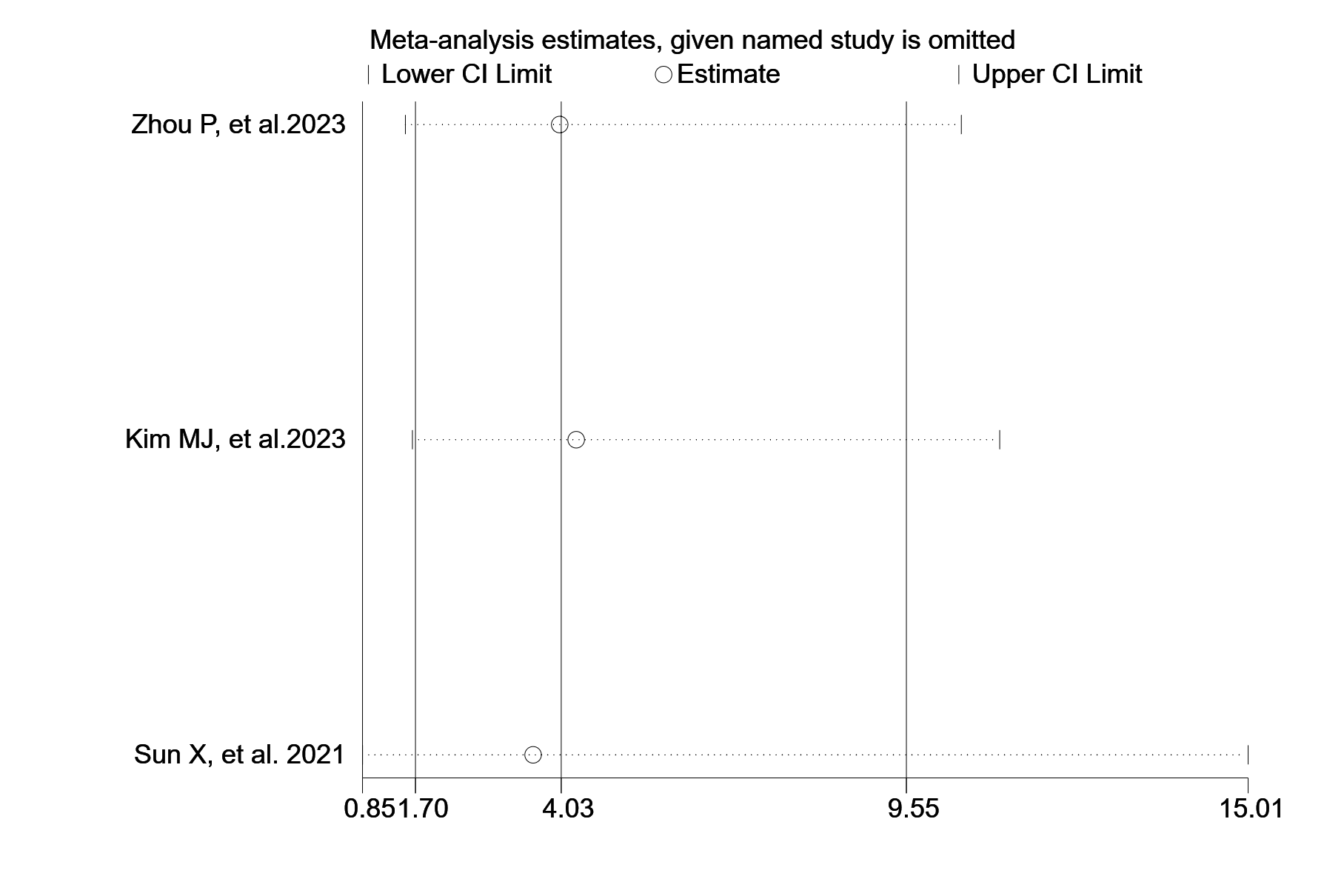


Supplementary Figure 8 Plot for the assessment of heterogeneity among the included studies of NI on poor prognosis in AAV-ILD through One-by-one elimination method


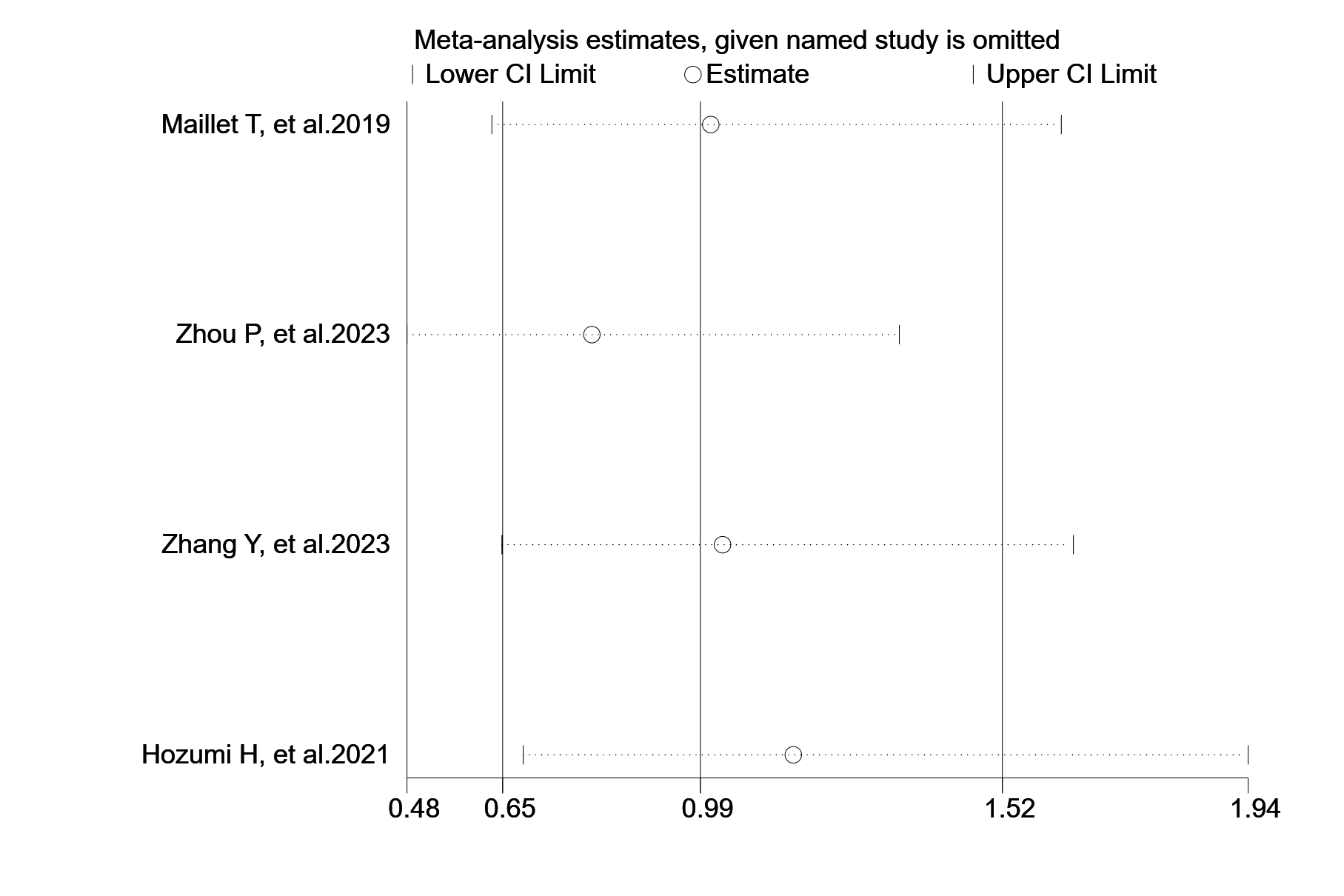


Supplementary Figure 9 Plot for the assessment of heterogeneity among the included studies of RI on poor prognosis in AAV-ILD through One-by-one elimination method


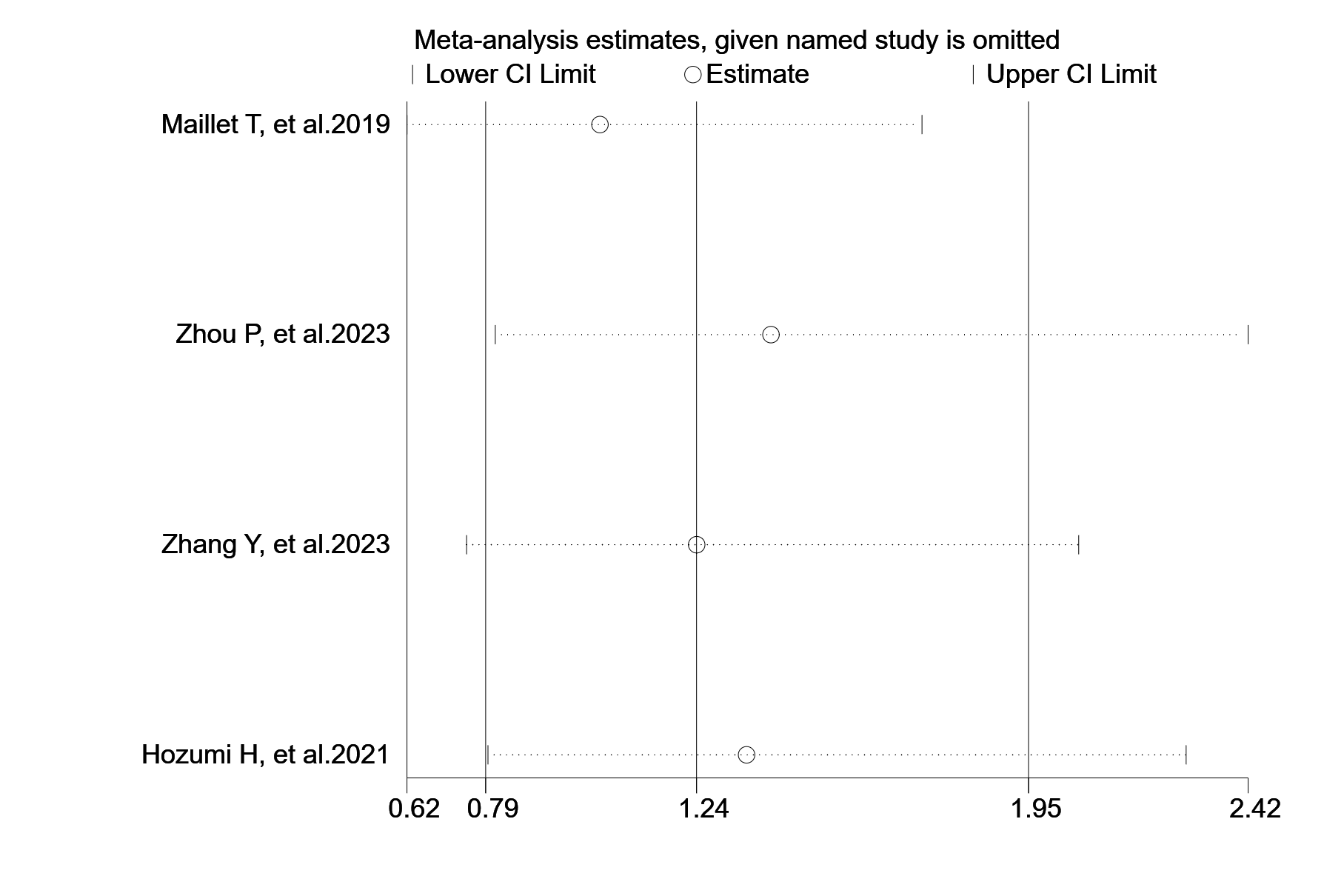


Supplementary Figure 10 Plot for the assessment of heterogeneity among the included studies of FFS score ≥ 1 on poor prognosis in AAV-ILD through One-by-one elimination method


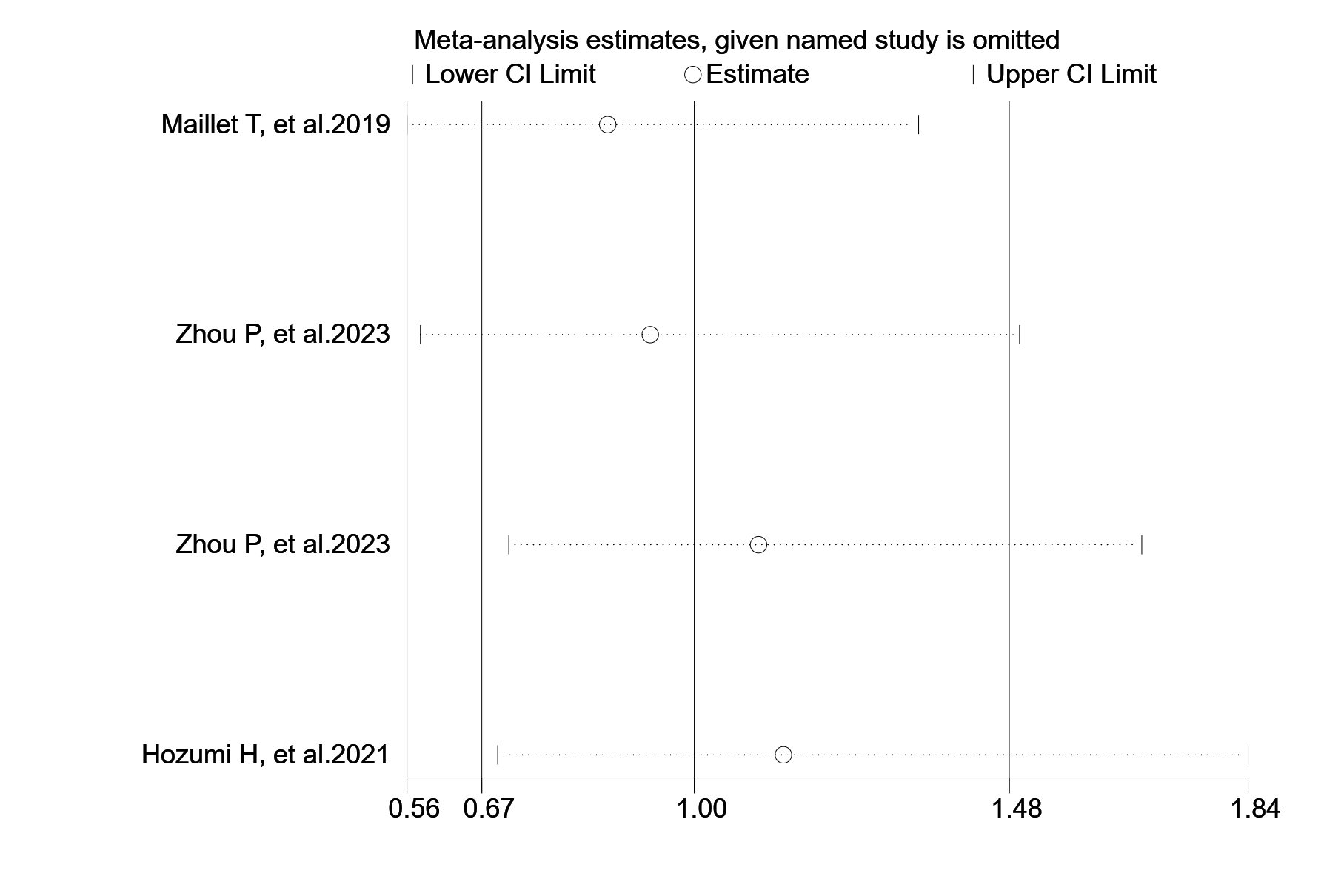


Supplementary Figure 11 Plot for the assessment of heterogeneity among the included studies of immunosuppressant for induction on poor prognosis in AAV-ILD through One-by-one elimination method


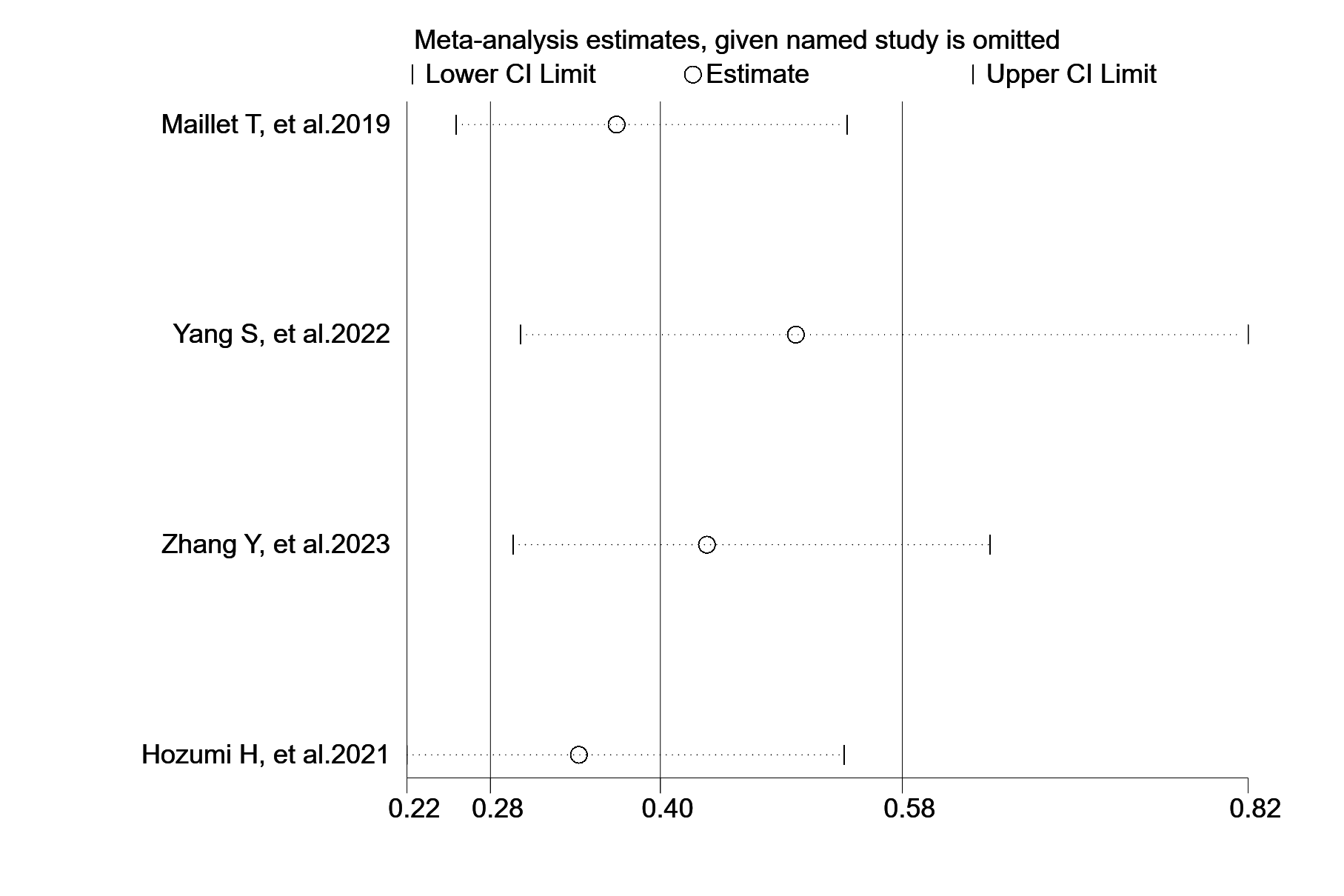

Supplement: Supplementary file 1 — Supplementary file1 (DOCX 69.3 MB) [file 10067_2025_7378_MOESM1_ESM.docx]
